# First Isolation of *Mycobacterium ulcerans* from an Aquatic Environment: The End of a 60-Year Search?

**DOI:** 10.1371/journal.pntd.0000216

**Published:** 2008-03-26

**Authors:** Tim Stinear, Paul D. R. Johnson

**Affiliations:** 1 Department of Microbiology, Monash University, Clayton, Victoria, Australia; 2 Department of Infectious Diseases, Austin Hospital, Heidelberg, Australia; Institut Pasteur, France

In a landmark paper in this issue of *PLoS Neglected Tropical Diseases*, Portaels et al. [1] describe the first isolation in pure culture of *Mycobacterium ulcerans* from an aquatic environment, ending a quest that began over 60 years ago when MacCallum and his Australian colleagues identified *M. ulcerans* as the causative agent of the ulcerative skin disease that later became known as Buruli ulcer [2]. This is a major achievement and will serve as the definitive reference point for scientists intent on revealing the ecology, environmental reservoir, and precise mode of transmission of *M. ulcerans*.

Buruli ulcer is a terrible, disfiguring disease of skin and soft tissue that may leave sufferers permanently disabled (Figure 1). Those most affected are children living in rural West and Central Africa, but the disease is known in more than 30 countries worldwide, and people of all ages and races are susceptible. In some highly endemic regions, Buruli ulcer is now more common than the two most notorious mycobacterial diseases, leprosy and tuberculosis (TB) [3].

**Figure 1:**
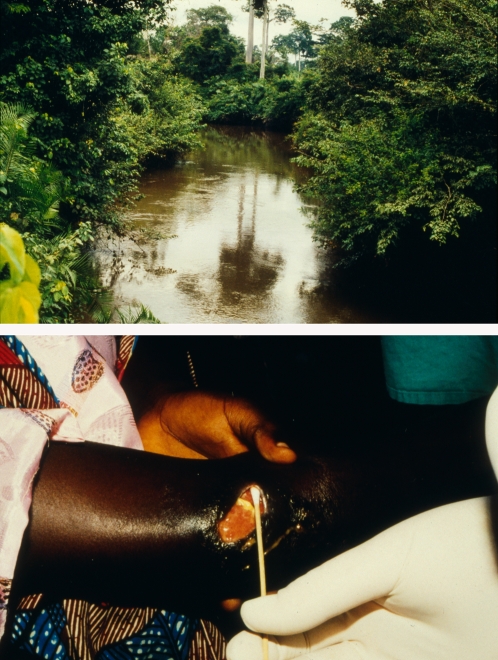
River Offin in the Amansie West District of Ghana showing a typical environmental setting for a region of Buruli ulcer endemicity, and *M. ulcerans* infection of the elbow of a female patient from the same district.

Recently, the combination of the potent antimycobacterial drugs rifampicin and streptomycin has been shown to be able to kill the causative agent, *M. ulcerans,* in early nodular Buruli ulcer [4], and a new WHO protocol has been implemented in several endemic countries [5]. The new protocol is proving very effective, greatly reducing costs and often avoiding the need for surgery [6]. More research is required if we are to understand and control this potentially devastating disease.

Exactly how *M. ulcerans* is introduced into the skin of humans remains unknown, but unlike TB or leprosy, the infection is acquired directly or indirectly from the environment and not from contact with other patients. The highly focal epidemiology and association with swamps and slow-flowing water are hallmarks of Buruli ulcer (Figure 1), and these observations have led to 50 years of failed attempts by many research groups to try to cultivate *M. ulcerans* from a variety of environmental sources that has included bats, sand flies, rodents, fish, molluscs, vegetation, water, and soil [7]–[11]. And while isolation of *M. ulcerans* from clinical specimens is relatively straightforward, efforts to isolate the bacterium from the environment have been confounded by its slow growth, its predicted paucity (based on PCR) in the environment, and the complex, competing microbial flora in these sample types.

The discovery of the *M. ulcerans*−specific insertion sequence IS*2404* in 1997 [12] and the subsequent development of molecular diagnostics for *M. ulcerans* was the catalyst for renewed efforts to find its environmental sources. The detection of IS*2404* in carnivorous water bugs indicated that aquatic insects (among other things) might harbour the bacterium [13]. Laboratory-based feeding studies with *M. ulcerans* in *Naucouris* sp. have shown that the bacterium can colonize the salivary glands of these insects and also be transferred by biting a mammalian (mouse) host to cause disease [14]. These observations led Portaels et al. to try and culture *M. ulcerans* directly from homogenates of five aquatic insects captured from a Buruli ulcer–endemic region of Benin. In a complicated and patience-testing process spanning more than 2 years and involving classical mycobacterial culture techniques and monitoring of cultures by IS*2404* PCR, followed by three rounds of blind passaging through mice and then further culture, the team were finally able to obtain a single *M. ulcerans* isolate from a water strider (*Gerris* sp.). Phenotypic analysis showed this isolate produced the same polyketide toxin as clinical isolates and was fully virulent for mice. Most importantly, molecular characterization confirmed it was *M. ulcerans* and not another recently reported *M. ulcerans*–like mycobacterium [15]. Molecular studies also revealed a novel single nucleotide polymorphism in the 16S rRNA gene of this strain, proving that the result was not a laboratory artefact caused by contamination. The same mutation has since been found in clinical *M. ulcerans* isolates recovered from patients in the same region of Benin, suggesting a common origin for environmental and patient isolates. Portaels and colleagues were the first to link insects with *M. ulcerans* [13], and in Australia, two recent papers have taken her observation one step further by demonstrating *M. ulcerans* DNA in mosquitoes and establishing that mosquito exposure increases the odds of Buruli ulcer in humans [16],[17]. However, whether insects transmit *M. ulcerans* to humans or just mark its presence in the environment has yet to be definitively established.

The research presented in this paper represents considerable technical prowess, and is the result of knowledge accrued over many decades of *M. ulcerans* research. It does not end the quest to establish the environmental source of *M. ulcerans* but provides encouragement (or perhaps discouragement depending on your natural disposition) and an opportunity for future studies to also attempt *M. ulcerans* isolation from the environment. It will only be through the analysis of many different *M. ulcerans* isolates from the environment, in the context of solid epidemiological and ecological data, that the lingering and critical questions surrounding the reservoirs and modes of transmission of *M. ulcerans* will finally be resolved. Much work remains to be done.

## References

[1] Portaels F, Meyers WM, Ablordey A, Castro AG, Chemlal K (2008). First cultivation and characterization of *Mycobacterium ulcerans* from the environment.. PLoS Negl Trop Dis.

[2] MacCallum P, Tolhurst J, Buckle G, HA S (1948). A new mycobacterial infection in man.. J Path Bacteriol.

[3] Debacker M, Aguiar J, Steunou C, Zinsou C, Meyers WM (2004). *Mycobacterium ulcerans* disease (Buruli ulcer) in rural hospital, Southern Benin, 1997–2001.. Emerg Infect Dis.

[4] Etuaful S, Carbonnelle B, Grosset J, Lucas S, Horsfield C (2005). Efficacy of the combination rifampin-streptomycin in preventing growth of *Mycobacterium ulcerans* in early lesions of Buruli ulcer in humans.. Antimicrob Agents Chemother.

[5] Anonymous (2006). Provisional guidelines for antibiotic treatment of Buruli ulcer..

[6] Chauty A, Ardant MF, Adeye A, Euverte H, Guédénon A (2007). Promising clinical efficacy of streptomycin-rifampin combination for treatment of Buruli ulcer (*Mycobacterium ulcerans* disease).. Antimicrob Agents Chemother.

[7] Barker DJP, Clancey JK, Rao SK (1972). Mycobacteria on vegetation in Uganda.. East Afr Med J.

[8] Buckle G (1972). Notes on *Mycobacterium ulcerans*.. Aust N Z J Surg.

[9] Portaels F (1973). [Environmental mycobacteria in Lower Zaire].. Ann Soc Belg Med Trop.

[10] Stanford JL, Paul RC (1973). A preliminary report on some studies of environmental mycobacteria.. Ann Soc Belg Med Trop.

[11] Ross BC, Johnson PD, Oppedisano F, Marino L, Sievers A (1997). Detection of *Mycobacterium ulcerans* in environmental samples during an outbreak of ulcerative disease.. Appl Environ Microbiol.

[12] Ross BC, Marino L, Oppedisano F, Edwards R, Robins-Browne RM (1997). Development of a PCR assay for rapid diagnosis of Mycobacterium ulcerans infection.. J Clin Microbiol.

[13] Portaels F, Elsen P, Guimares-Peres A, Fonteyne PA, Meyers WM (1999). Insects in the transmission of *Mycobacterium ulcerans* infection [letter].. Lancet.

[14] Marsollier L, Robert R, Aubry J, Saint Andre JP, Kouakou H (2002). Aquatic insects as a vector for *Mycobacterium ulcerans*.. Appl Environ Microbiol.

[15] Yip MJ, Porter JL, Fyfe JA, Lavender CJ, Portaels F (2007). Evolution of *Mycobacterium ulcerans* and other mycolactone-producing mycobacteria from a common *Mycobacterium marinum* progenitor.. J Bacteriol.

[16] Johnson PD, Azuolas J, Lavender CJ, Wishart E, Stinear TP (2007). *Mycobacterium ulcerans* in mosquitoes captured during outbreak of Buruli ulcer, southeastern Australia.. Emerg Infect Dis.

[17] Quek TY, Athan E, Henry MJ, Pasco JA, Redden-Hoare J (2007). Risk factors for *Mycobacterium ulcerans* infection, southeastern Australia.. Emerg Infect Dis.

